# Clinical Significance and Prognostic Value of TLR4 and AGER in Inflammatory Breast Cancer

**DOI:** 10.3390/cancers17132182

**Published:** 2025-06-28

**Authors:** Luiza Darla Aguiar Silva Paiva, Ana Carolina Filgueiras Teles, Jeferson dos Santos Souza, Pedro Ruan Amorim Oliveira, Bianca Elen Souza Alves, Mariana Timbaúba Benício Coelho, Aurilene Gomes Cajado, Isabelle Fátima Vieira Camelo Maia, Paulo Goberlânio Barros Silva, Diane Isabelle Magno Cavalcante, Maria do Perpétuo Socorro Saldanha Cunha, Larissa Mont’Alverne Arruda, Roberto César Pereira Lima-Júnior, Silvia Regina Rogatto, Deysi Viviana Tenazoa Wong

**Affiliations:** 1 Graduate Program in Oncology, Haroldo Juaçaba Hospital, Cancer Institute of Ceará (ICC), Fortaleza 60430-230, CE, Brazilpaulo.goberlanio@gmail.com (P.G.B.S.); dra_lari2004@yahoo.com.br (L.M.A.); robertocesar@ufc.br (R.C.P.L.-J.); 2 Laboratory of Inflammation and Cancer Pharmacology, Drug Research and Development Center (NPDM), Department of Physiology and Pharmacology, Faculty of Medicine, Federal University of Ceara, Fortaleza 60430-270, CE, Brazildiane.cavalcante@ufc.br (D.I.M.C.); 3 Health Technology Institute, SENAI CIMATEC, Salvador 41650-010, BA, Brazil; 4 Graduate Program in Pathology, Faculty of Medicine, Federal University of Ceara, Fortaleza 60430-160, CE, Brazil; 5 Department of Clinical Genetics, University Hospital of Southern Denmark, 7100 Vejle, Denmark; silvia.rogatto@unesp.br; 6 Botucatu Medical School Hospital, São Paulo State University—UNESP, Botucatu 18618-687, SP, Brazil

**Keywords:** TLR4, AGER, obesity, inflammatory breast carcinoma

## Abstract

**Background/Objectives**: Inflammatory breast carcinoma (IBC) is an aggressive and rare neoplasm, accounting for 1–5% of all breast cancers. Toll-like receptor type 4 (TLR4) and Advanced Glycation End Products Receptor (AGER/RAGE) have been implicated in breast cancer, and have been shown to promote tumor growth, metastasis, and resistance to therapy by modulating the tumor microenvironment and inflammatory pathways. However, the role of TLR4 and AGER in IBC has not been elucidated. **Methods**: TLR4 and AGER immunofluorescence expression were evaluated in 27 IBC and 24 non-IBC samples. The expression data and clinicopathological parameters, including the prognostic values of these biomarkers, were compared. *TLR4* and *AGER* gene expression were investigated using the microarray transcriptomic dataset of IBC and non-IBC samples (Gene Expression Omnibus repository—GEO). **Results**: IBC samples showed higher TLR4 and AGER immunoexpression than the non-IBC group and were associated with obesity and Ki-67 expression (*p* < 0.05). AGER expression in IBC versus non-IBC was also statistically associated with triple-negative molecular subtypes. Non-IBC subjects with AGER immunoexpression above the cutoff (106.1%, sensitivity of 92.3%, and specificity of 56.2%) showed reduced metastasis-free survival (*p* = 0.032). In the multivariate analysis, high TLR4 immunostaining increased the risk of metastasis-free survival by 1.029-fold. Analyzing three external GEO datasets confirmed that *TLR4* and *AGER* expression increased in IBC compared to non-IBC samples. **Conclusions**: Overall, IBC samples showed higher TLR4 and AGER expressions than other breast cancer types, shedding light on the significance of these markers on IBC biology.

## Simple Summary

Inflammatory breast cancer is a rare and aggressive form of breast cancer, making it crucial to understand what drives its growth. This study focuses on two proteins, TLR4 and AGER, which are known to play roles in other types of breast cancer but have not been well-studied in inflammatory breast cancer. We want to find out if these proteins are more active in inflammatory breast cancer compared to other breast cancers and if they relate to how the cancer behaves. By studying these proteins, we aim to uncover new insights that may ultimately lead to improved methods for diagnosing, treating, and managing this complex disease. Understanding the roles of TLR4 and AGER could offer new targets for future therapies and improve outcomes for patients with inflammatory breast cancer.

## 1. Introduction

Breast cancer (BC) is the most common tumor type among female adults, and in 95% of countries, BC is the first cause of death in women [1]. In Brazil, BC shows a similar epidemiological pattern. Excluding non-melanoma skin tumors, BC ranks first in incidence, with an estimated 73,000 new cases each year from 2023 to 2025 [2]. It corresponds to an age-adjusted estimated risk of 41.89 cases per 100,000 inhabitants. In 2020, the number of deaths from this neoplasm was 17,825, making it the leading cause of cancer-related death among women in the country [3].

Inflammatory breast carcinoma (IBC) is a rare BC subtype, accounting for 1% to 10% of all BC cases [4]. It is the most aggressive and deadly form of BC, with almost twice the deaths when compared to locally advanced BC [5,6]. IBC patients have rapid progression early metastasis [7].

The diagnostic criteria for IBC include classic signs of inflammation, such as erythema, occupying at least one-third of the breast, edema, and warmth, with or without an underlying palpable mass. In addition, there is a rapid onset, generally less than six months, and pathological confirmation of invasive carcinoma [8]. A higher incidence of IBC has been described in African Americans, women at younger ages, and those with a high body mass index (BMI). Obesity significantly increases the risk of IBC regardless of hormone receptor status [9]. Obesity is characterized by inflammatory components and increased circulating levels of pro-inflammatory proteins, which impair immune function and cell-mediated immune responses [10].

The identification of tumor aggressiveness markers is an unmet need. The expression of Ki-67, a proliferative cell marker, is significantly higher in breast tumors where numerous viral DNAs are detected [11]. Additionally, multiple viral DNAs are more frequently observed in IBC samples than in non-IBC samples, likely contributing to disease progression [11]. The pathogenic properties of IBCs are associated with the overexpression of the translation initiation factor eIF4GI in most IBCs, which contributes to tumor cell survival and the formation of tumor emboli [12]. Notably, the interplay between products of cell death and the activation of the immune response is driven by pattern recognition receptors, including toll-like receptor 4 (TLR4) [13,14] and advanced glycation end products receptor (AGER/RAGE) [15]. Remarkably, TLR4 and AGER share a common ligand, the high-mobility group box 1 (HMGB1) [15,16], a damage-associated molecule extensively implicated in cancer pathogenesis [17,18,19]. However, the role of TLR4 and AGER, specifically in inflammatory breast carcinomas, has not been fully elucidated. Since HMGB1 is overexpressed in breast cancer, TLR4 and AGER assessment offers a broader mechanistic perspective into innate immune activation and damage-associated inflammation, reinforcing the rationale for focusing on these markers.

TLR4 signals through the adaptor protein MyD88 and contributes to insulin resistance [20] as well as cancer cell invasion and metastasis [21]. It is estimated that 20% of mononuclear inflammatory cells express TLR4 in the breast tumor microenvironment, which is associated with tumor aggressiveness [22]. Breast cancer biopsies present higher expression levels of TLR4 compared to normal breast tissues [23].

The transmembrane protein AGER is a key player in innate immunity and inflammation process. It is responsible for recognizing damage-associated molecular patterns and has the potential to trigger chronic inflammatory processes, leading to conditions such as diabetes, obesity, and cancer [15]. In BC, increased AGER expression was reported at advanced stages [24], sparking further interest in its role in disease progression. A study carried out in northeastern China showed that patients with the AGER gene polymorphism have a 1.6-fold increased risk of developing breast cancer [25].

In this study, we evaluated the expression of TLR4 and AGER in inflammatory and non-inflammatory breast carcinoma samples, comparing the findings with clinical and histopathological data and their prognostic values. Further analysis included the expression pattern of these molecules using public transcriptomic data from the IBC and non-IBC groups in the Gene Expression Omnibus repository.

## 2. Materials and Methods

### 2.1. Study Design and Ethics

This retrospective, cross-sectional, case-control study was conducted at the Haroldo Juaçaba Hospital, Ceará Cancer Institute (ICC), Brazil. Our cohort consisted of patients who were diagnosed with IBC or non-IBC from 2017 to 2022. This study was conducted in accordance with human research advisory standards and Good Clinical Practice (GCP) guidelines and was approved by the institutional Human Research Ethics Committee (Approval Protocol # 5.010.710).

Tumor and adjacent non-tumor specimens used in this study were obtained from the Department of Pathology at the Cancer Institute of Ceará, which is legally authorized as a tumor depository and possesses the required informed consent and authorization documents for research use of the samples.

### 2.2. Subject and Data Collection

Inclusion criteria were as follows: (1) patients with a clinical diagnosis of IBC, defined as T4d tumors presenting inflammatory signs of the breast, such as erythema, edema, or peau d’orange appearance; (2) adequate and well-fixed core biopsy (diagnostic) tumor samples for analysis; and (3) archived formalin-fixed, paraffin-embedded (FFPE) tissue samples. Exclusion criteria comprised the following: (1) samples unsuitable for sectioning due to insufficient size or poor fixation, compromising immunohistochemistry (IHC) and immunofluorescence (IF) analysis, and (2) cases with suspected or confirmed mastitis or other differential diagnoses of IBC. Around 500 records with a suspected diagnosis of IBC were screened and individually re-analyzed. All cases were selected based on the IBC classification criteria presented in the American Joint Committee on Cancer 8th Edition (AJCC 2018).

Sample size estimation was based on historical data from the oncology service involved in the study. We estimated that approximately 5% of patients diagnosed with primary BC and receiving neoadjuvant chemotherapy would present with the inflammatory phenotype. Given the prevalence of IBC, the initial sample size calculation—using a two-sided test for independent means—assumed a minimum detectable difference (Δ) of 29% in marker expression between groups, a significance level of 5% (α = 0.05), and 80% statistical power (1 − β = 0.80) [26]. This calculation indicated a requirement of 31 participants per group.

Considering the tumor rarity and sample loss during assay processing, we eventually included samples from 27 IBC and 24 non-IBC patients; all of them were untreated before surgery. Samples of the primary tumor, surrounding non-tumor tissue, and emboli were collected from these patients. We found tumor emboli in both groups; however, IBC showed dermal embolization. It is worth noting that the presence of emboli is not a diagnostic criterion of IBC.

The presence of a swollen or hyperemic-involved breast, nipple alteration, and diffuse skin alteration was clinically confirmed for inclusion as IBC in addition to tumor size T4d. We selected non-IBC samples characterized as breast tumors at clinical stages IIIA, IIIB, IIIC, or IV with the absence of an inflammatory phenotype.

Sociodemographic, clinical, and pathological variables were analyzed, including age at diagnosis, family history of cancer, obesity, staging, estrogen receptor (ER) status, progesterone receptor (PR) status, human epidermal growth factor receptor 2 (HER2) status, cell proliferation index (Ki-67), and local and distant recurrence. The World Health Organization (WHO) criteria were used to assess obesity based on body mass index (BMI = weight/height^2^), with patients considered to have obesity if their BMI was 30 kg/m^2^ or higher.

### 2.3. TLR4 and AGER Immunofluorescence

The formalin-fixed, paraffin-embedded tissue sections were deparaffinized, hydrated, and washed under tap water and phosphate-buffered saline (PBS). The antigenic recovery was performed using 0.1 M sodium citrate buffer (pH 6.0) at 95 °C, followed by permeabilization with 0.1% Triton X-100 (Sigma-Aldrich, St. Louis, MO, USA). Tissue sections were incubated with a solution containing 0.3 M glycine (Sigma-Aldrich^®^, St. Louis, MO, USA) and 5% bovine serum albumin (BSA) (Sigma-Aldrich^®^, St. Louis, MO, USA). Next, the tissues were incubated overnight at 2–8 °C with the mouse monoclonal primary antibody anti-TLR4 (1:200, Cat# sc-293072, RRID: AB_10611320, Santa Cruz Biotechnology, Inc., Dallas, TX, USA) or rabbit polyclonal primary antibody anti-AGER (1:50, Cat# PA5-24787, RRID: AB_2542287, Invitrogen, Thermo Fisher Scientific^®^, Waltham, MA, USA), followed by the secondary antibody donkey anti-mouse IgG Alexa-568 (1:200) and goat anti-mouse IgG Alexa Fluor 568, respectively (1:400, Thermo Fisher Scientific, Waltham, MA, USA), for 1.5 h. Tissues were exposed to a 0.002% DAPI (4,6′-diamidino-2-phenylindole) /PBS solution (Thermo Fisher Scientific) for 30 min for cell nucleus staining. ProLong Gold Antifade Mountant (Thermo Fisher Scientific) mounted the slides. The photomicrographs were obtained using an immunofluorescence microscope (Agilent Biotek Cytation 3, Santa Clara, CA, USA), with standardization of master gain and digital offset for analysis. The fluorescent intensity was quantified using image software (Image J version 1.54g, RRID: SCR_002285, National Institutes of Health, Washington, DC, USA).

### 2.4. TLR4 and AGER Gene Expression Analysis

We selected IBC and non-IBC microarray transcriptomic datasets from the Gene Expression Omnibus (GEO) repository to investigate the expression of TLR4, MyD88, and AGER genes. The GEO databases had accession codes GSE45581 [27] and GSE111477 [28]. The GSE111477 dataset, also included, was deposited on two different microarray platforms: GPL5188 (Affymetrix transcript-genes) and GPL5175 (Affymetrix probe set-exons). Data were collected using the GEOquery package v.2.70.0 [29] and analyzed using the R programming language v.4.3.1. Data were normalized by log2 using the limma package version 3.58.1 [30]. To consider probes with statistical significance, a hypothesis test (*t*-test) was performed using the matrix tests package version 0.2.3 [31], employing the row_t_welch method. A statistical filter with *p*-value < 0.05 was assigned to obtain the probes with statistical significance. Probes that were not statistically significant were excluded. Genes presenting more than one probe were grouped by the averages of all selected probes. Boxplot graphs were generated using the ggplot2 package version 3.5.0 [32].

### 2.5. Statistical Analysis

Statistical analysis was performed using GraphPad Prism^®^ software version 8.0 and IBM SPSS Statistics version 20.0. Clinicopathological characteristics of IBC and non-IBC patients were analyzed using the Chi-square or Fisher’s exact test. The results were expressed as mean ± SD (standard deviation) for fluorescence intensity. The Kolmogorov–Smirnov test was conducted to assess the normality of the variables. Two-way ANOVA and the Mann–Whitney test were used to compare the expression of TLR4 and AGER in IBC and non-IBC samples. The receiver operating characteristic (ROC) curve and area under the curve (AUC) were used to determine the cutoff point of the markers analyzed by immunofluorescence to estimate their prognostic values. The cutoff expression values were determined using the Youden index (J = sensitivity + specificity − 1). We used the log-rank test to analyze metastasis-free survival (the period from the start of treatment for cancer when a patient is still alive and cancer has not spread to other parts of the body). Additionally, a Cox regression model was used for multivariate analysis of metastasis. A *p*-value ≤ 0.05 was considered significant.

## 3. Results

Clinicopathological characteristics of IBC and non-IBC patients are detailed in Table 1. Our cohort comprised 27 women with IBC and 24 non-IBC women, respectively, with a median age of 55 (range, 36–83) and 57 (range, 32–91). The IBC patients presented comparable patterns of obesity; lymph node involvement; metastasis; expression levels of ER, PR, HER, and Ki-67 above 20%; and the number of triple-negative breast cancers (TNBCs) (*p* > 0.05).

**Figure 1:**
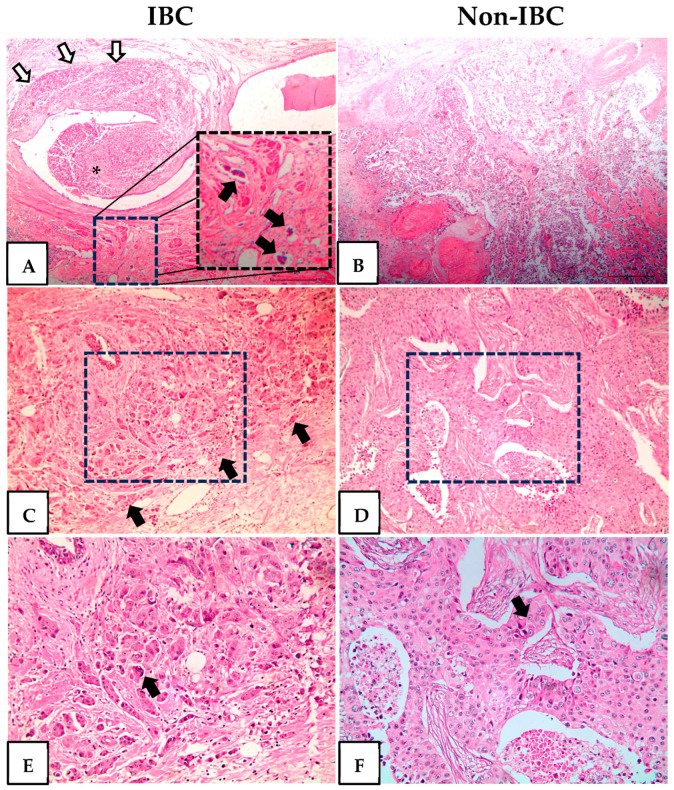
Hematoxylin and eosin staining of inflammatory breast cancer (IBC) and non-IBC tissue sections. Representative photomicrographs of IBC and non-IBC. (**A**) Invasive carcinoma (white arrows) surrounds and protrudes in the lumen of a lactiferous duct (asterisk). At the bottom are three dilated lymphatic vessels with tumor emboli. The dotted square is shown at a higher magnification at the right of the same panel and the tumor emboli indicated by black arrows. (**B**) Invasive carcinoma, high grade, with necrosis. (**C**) Infiltrative small nets of high-grade invasive carcinoma (black arrows). The area delimitated by the dotted square is shown at a higher magnification in panel (**E**). (**D**) Invasive carcinoma with a solid pattern. The area delimitated by the dotted square is shown at a higher magnification in panel (**F**). (**E**) Pleomorphic, grade 3 nuclei (black arrows). (**F**) Sheets of cells with enlarged nuclei with prominent nucleoli (black arrows). Original magnification H&E-stained slides 40× (**A**,**B**) 100× (**C**,**D**) and 200× (**E**,**F**).

Histopathological analysis of IBC revealed invasive high-grade carcinomas with dilated lymphatic vessels infiltrated by tumor emboli, small infiltrative carcinoma nests, and pleomorphic nuclei (Figure 1A,C,E). All non-IBC cases were high-grade invasive carcinomas characterized by necrosis and sheets of cells with enlarged and prominent nucleoli (Figure 1B,D,F).

**Figure 2:**
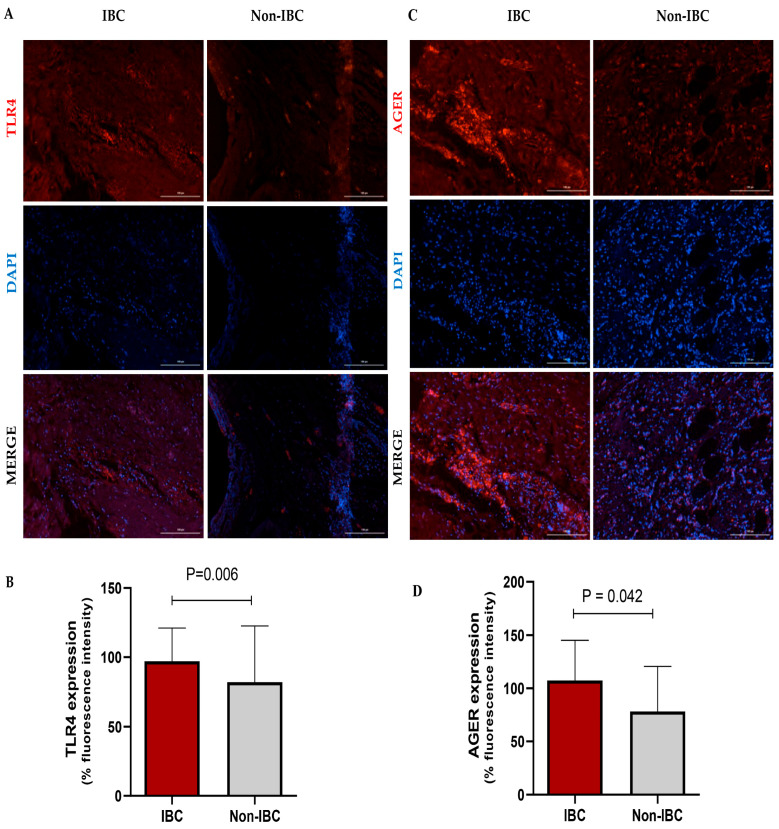
TLR4 and AGER expression analysis in inflammatory breast cancer (IBC) and non-IBC patients. Representative photomicrographs show the distribution of TLR4 (**A**,**B**) and AGER (**C**,**D**) protein expression in IBC and non-IBC samples. Red, TLR4 staining or AGER staining; blue, DAPI nuclear staining. The scale bar represents 100 µm, with an original magnification of 200×. (**B**) The quantification of immunofluorescence intensity expressed as a percentage. TLR4 and AGER expression in IBC samples is higher than in non-IBC. Data are expressed as the mean ± SD, and the statistical analysis was performed with the Mann–Whitney test.

**Figure 3:**
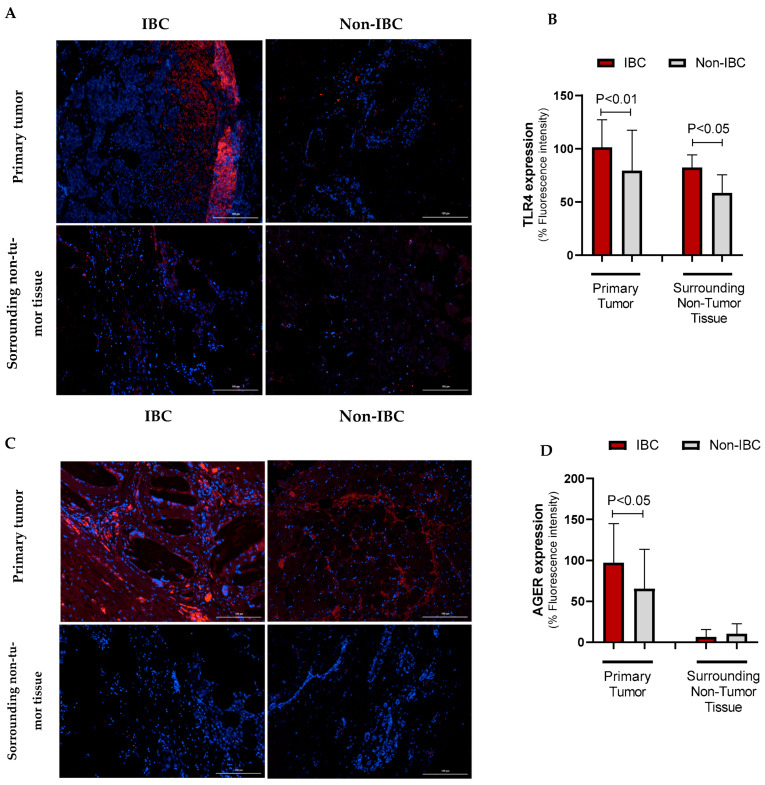
TLR4 and AGER immunofluorescence intensity in IBC and non-IBC samples and surrounding non-tumor tissues. Representative images are depicted in (TLR4: (**A**), AGER: (**C**)), while the data from the quantification of the TLR4 and AGER fluorescence intensity is shown in (**B**,**D**). Data is expressed as the mean ± SD. The statistical analysis was conducted with the Mann–Whitney test. The scale bar represents 100 µm.

The expression of TLR4 and AGER proteins was investigated using immunofluorescence assays in IBC and non-IBC samples (Figure 2A–D, respectively). TLR4 expression was significantly higher (*p* = 0.006) in IBC (97.1 ± 24.0) than in non-IBC samples (82.1 ± 40.6) (Figure 2A,B). We also analyzed TLR4 expression in the surrounding non-tumor tissue. Notably, IBC samples showed higher intensity of TLR4 expression not only in the tumors but also in surrounding non-tumor tissues (92.4 ± 18.5) compared with surrounding non-tumor tissues from the non-IBC group (61.2 ± 18.7; *p* < 0.05) (Figure 3A,B). Similarly to TLR4, AGER immunofluorescence showed an increased expression (107.4 ± 37.9) in the IBC group when compared to non-IBC samples (78.4 ± 42.2) (*p* = 0.042) (Figure 2C,D and Figure 3C,D).

The higher immunostaining of TLR4 in IBC samples was associated with Ki-67 ≥ 20% (Table 2). The expression of TLR4 was also significantly increased in IBC cases, as indicated by the Ki-67 proliferation index, which contrasted with the predominant low Ki-67 expression in the non-IBC group (*p* = 0.002).

The percentage of AGER expression (mean 133.8 ± 33.0) was significantly increased (*p* = 0.05) in IBC patients with subjects with obesity compared to non-IBC individuals with no obesity (mean 86.6 ± 40.9). Additionally, triple-negative IBC patients (i.e., negative for ER, PR, and HER2) presented increased AGER expression (136.8 ± 6.8) compared to subjects with other BC phenotypes in non-IBC samples (87.7 ± 40.3) (*p* = 0.045). Moreover, IBC samples had an increased AGER expression compared to non-IBC (121.1 ± 36.5 vs. 64.2 ± 33.5) cases according to Ki-67 above 20% (*p* = 0.049) (Table 2).

**Figure 4:**
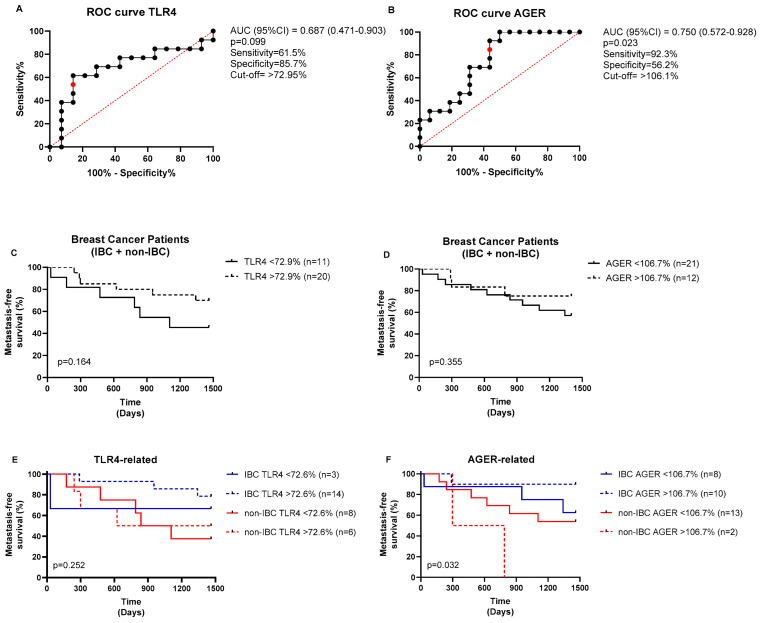
ROC curves for TLR4 and AGER expressions and their prognostic values. ROC curves for (**A**) TLR4 and (**B**) AGER. The optimal cutoff values (red points) were determined from the cases analyzed (black dots) using the Youden index (J = sensitivity + specificity − 1). The analysis of the metastasis-free survival of BC patients stratified based on (**C**) TLR4 and (**D**) AGER cutoff expressions. The prognostic values of (**E**) TLR4 and (**F**) AGER expressions were also analyzed in IBC and non-IBC subjects. The log-rank test was used to compare the survival between the groups.

The ROC (receiving operating characteristic) curve analysis was performed to determine the area under the curve (AUC), sensitivity, and specificity, thereby obtaining the fluorescence cutoff for each protein (low or high expression). As shown in Figure 4, the cutoff for TLR4 fluorescence intensity was 72.95%, yielding a sensitivity of 61.5% and a specificity of 85.7%, with no statistical significance found (*p* = 0.099, Figure 4A). The cutoff for AGER immunostaining was 106.1%, yielding a sensitivity of 92.3% and a specificity of 56.2% (*p* = 0.023, Figure 4B). The BC patients stratified based on the cutoff points of each marker revealed no impact on metastasis-free survival (*p* > 0.05, Figure 4C,D). Conversely, the high-AGER-expressing group had significantly shorter metastasis-free survival than the other groups (*p* = 0.032, Figure 4F), whereas TLR4 did not affect the patients’ survival (*p* > 0.05, Figure 4E). In the multivariate analysis (Table 3), radical mastectomy reduced the risk of metastasis by 0.022-fold, high TLR4 immunostaining increased the risk by 1.029-fold, and hormone therapy reduced the risk by 0.034-fold, independent of other variables in the model.

**Figure 5:**
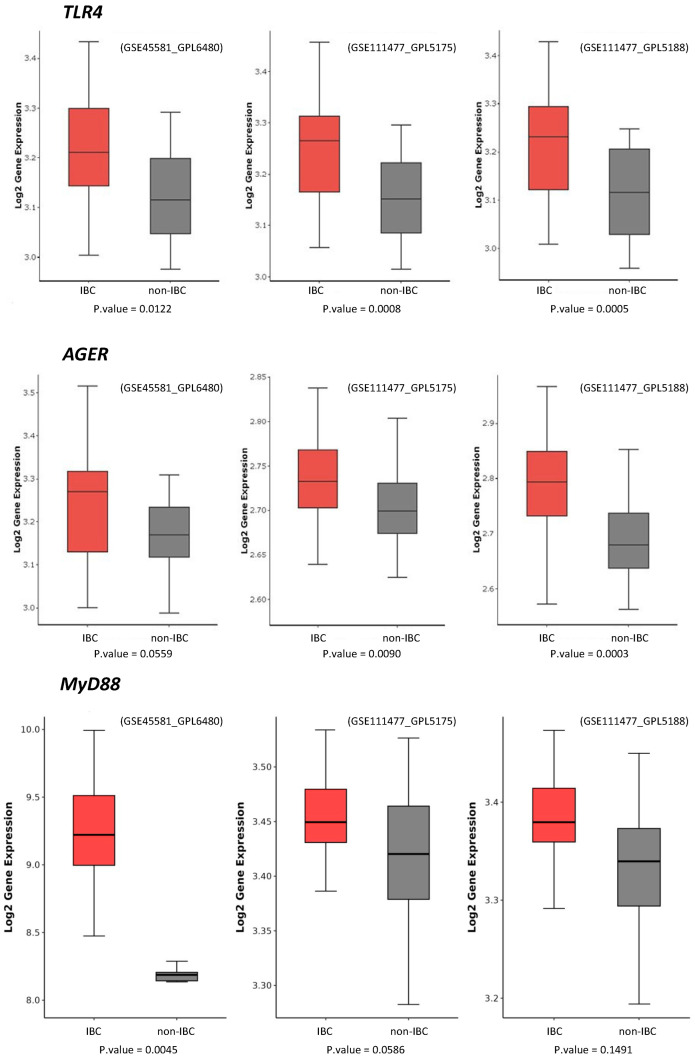
TLR4, MyD88, and AGER gene expression in inflammatory breast cancer (IBC) and non-IBC cases using GEO datasets. Data were collected using the GEOquery package version 2.70.0 and analyzed using the R programming language. Data were normalized by log2. A statistical filter with *p*-value < 0.05 was assigned to obtain the probes with statistical significance. Probes that were not statistically significant were excluded. Boxplot graphs were generated using the ggplot2.

We further compared the expression of TLR4 and AGER proteins with three external datasets of IBC and non-IBC samples evaluated by microarray expression analysis (GSE45581_GPL6480, GSE111477_GPL5175, and GSE111477_GPL5188). These three studies confirmed the higher expression of TLR4 and AGER genes in IBC samples compared to non-IBC samples (Figure 5). These external datasets also revealed an increased gene expression of *MyD88*, a downstream adaptor protein in TLR4 signaling (Figure 5).

## 4. Discussion

The relationship among TLR4, AGER, and breast cancer has been documented in BC cell lines and tissue samples [21,23,33,34]. The activation of the TLR4 and AGER pathways in MCF-7 and MDA-MB-231 breast cancer cell lines promotes β-catenin signaling pathway-activated cell migration. It enhances cell proliferation through the PI3K/AKT/GSK3β pathway, contributing to breast cancer (BC) metastasis [23,33,34]. Increased TLR4 gene expression has been reported in BC samples compared to normal breast tissues. In addition, the authors evidenced that PAMPs, such as LPS, activate the TLR4 pathway in breast cancer cell lines [23]. However, the impact of the differential expression of TLR4 and AGER on the clinical presentations of BC, such as inflammatory breast cancer (IBC), has not yet been investigated. While the role of these proteins in other BC types may suggest their potential upregulation in IBC, our study provides experimental confirmation of this hypothesis using patient-derived samples. Importantly, our immunofluorescence data objectively quantified the differential protein expression levels in IBC samples compared to non-IBC, supporting the proposed link between TLR4, AGER, and IBC pathogenesis.

Although IBC frequently presented with higher grades and enrichment of hormone receptor-negative, HER2-positive, or triple-negative tumors, we found no statistical difference in comparing the clinicopathological parameters between the IBC and non-IBC groups. This finding could be explained based on the limited number of cases evaluated and/or the selection criteria, in which the cases showed more homogeneous phenotypes.

A recent meta-analysis of BC assessed the correlation between TLR4 and clinicopathological parameters as well as survival outcomes. Increased TLR4 expression was associated with lymph node metastasis, tumor size (≥2 cm), PR expression, clinical stage, and shorter disease-free survival; however, it was not associated with histological grade, ER expression, or HER-2 status [35]. In our study, the lack of statistical significance between TLR4 expression and most clinicopathological parameters appears to contradict the meta-analysis results. However, our study was specially conducted in locally advanced and metastatic BC cases, suggesting that TLR4 might provide a differential contribution to BC pathogenesis depending on the disease’s advanced stages. We found a significant association between TLR4 and obesity in IBC subjects as well as a positive correlation with Ki-67 expression when comparing IBC versus non-IBC cases.

In inflammatory breast cancer, obesity contributes as a risk factor [36]. It is suggested that overweight or obese women are more likely to develop aggressive tumors [37]. We found that 28% of IBC patients had a high BMI, and 81.5% had an abnormal proliferative rate (Ki-67 > 20%), which is knowingly associated with a worse prognosis. High BMI differentially affects premenopausal and postmenopausal women, which can be explained by a negative feedback mechanism on the hypothalamic–pituitary axis, reducing gonadotropin release from high estrogen levels produced by the ovary and peripheral tissue [38]. The mechanism is not fully understood, but adipose tissue releases resistin to promote epithelial–mesenchymal transition, tumorigenesis, and metastasis through a TLR4 signaling pathway [39]. Additionally, insulin growth factor-1 (IGF-1)-dependent activation of the AGER signaling in adjacent endothelial cells fuels angiocrine mechanisms and tumor progression [40].

We did not investigate the underlying TLR4- and AGER-driven mechanisms; however, we consistently demonstrated that IBC predominantly expresses high levels of these markers in contrast to non-IBC cases. By stratifying IBC cases according to Ki-67 above 20%, we found a significantly increased expression of TLR4 and AGER. Ki-67 is of paramount importance for the clinical management of cancer. Patients with luminal breast cancer with Ki-67 below 14% and intermediate between 14 and 19% had the same disease-free interval and disease-specific survival and a better prognosis when compared to patients with Ki-67 above 20% [41]. More advanced tumor stages and nodal status are associated with high percentages of Ki-67, and therefore, the more aggressive the tumor is, the higher the Ki-67 [42]. It partially explains the proliferative IBC phenotype. Although no significant association was found between TLR4 and AGER expression and lymph node involvement, we detected a marginal significance of these markers in IBC compared to non-IBC subjects.

Among our IBC cases, 22.2% were diagnosed as triple negative. The impact of the aggressive phenotype and worse prognosis in IBC classified as triple-negative tumors has been demonstrated [43]. The overall survival time is expected to be 33 months for the pooled analysis of all IBC molecular subtypes, i.e., ER+/PR+, HER2 negative, ER+HER2+, HER2+, and triple-negative cases. Conversely, triple-negative IBC patients present a lower survival (<24 months) rate when compared to all other BC subtypes [43].

Interestingly, we showed increased AGER expression in triple-negative IBC compared to non-IBC samples, which might contribute to the worse prognosis frequently described in these tumor types. Notably, AGER expression above the cutoff of 106.7% presented reduced metastatic-free survival, particularly in non-IBC patients, suggesting it could differentially contribute to a worse prognosis depending on the breast cancer subtype. AGER promotes tumor progression and metastasis-related pathways, particularly in aggressive subtypes like triple-negative breast cancer [44], making it a candidate for risk stratification. A large cohort of 1904 breast cancer patients from the METABRIC study demonstrated that AGER and the insulin receptor are co-expressed and associated with a worse prognosis [45]. AGER gene expression is higher in BC stages II–III, and lower overall survival is observed in cases with high AGER expression [45]. Based on those findings, it was demonstrated that AGER inhibitors have attenuated metastasis development in murine TNBC models [46]. Additionally, results from the proteomic analysis of serum from tumor-bearing mice indicate that AGER inhibition affects metastatic mechanisms by decreasing the expression of STAT3 and AKT [46]. Future studies should validate AGER’s utility in guiding patient management, including the use of targeted therapies.

The differential TLR4 expression in IBC and non-IBC samples was further investigated in the surrounding tissue. We demonstrated a significant increase in TLR4 immunostaining on IBC cells and adjacent non-tumor tissues. Immune and inflammatory cells can communicate via paracrine interactions through cytokine secretion with the tumor microenvironment in the context of IBC [47]. The cytokines orchestrate the accumulation and effector mechanisms of tumor-infiltrating lymphocytes or tumor-associated macrophages [47]. TLR4 overexpression in tumors is often associated with chemoresistance to paclitaxel, a drug used in neoadjuvant chemotherapy regimens for breast cancer and tumor growth [48]. Since TLR4 expression was found in tumor cells and adjacent non-tumor tissues, mainly in IBC samples, its functional significance merits validation. Reinforcing the pathogenic role of TLR4, a multivariate analysis indicated that high TLR4 expression was implicated with a poor prognosis, favoring a metastatic risk.

While this study provides insights into specific marker expression patterns in IBC, some limitations must be acknowledged. The relatively small sample size in our study, based on the rarity of IBC (5% prevalence), resulted in 71% power, which is slightly below conventional thresholds and may constrain some of the assumptions of our findings. The reduced power to detect variations in marker expressions was further compromised by selection bias from a single tertiary center, sample loss during assay processing, and variations in analytical techniques that may affect the external validity of the results. Despite the statistically significant differences observed in the present study, biological validation in larger, multicenter cohorts with standardized phenotyping protocols is warranted.

Our study provides novel insights into the significance of TLR4 and AGER in inflammatory breast cancer. While it remains unclear whether these receptors function in a compensatory or complementary manner in IBC samples, the fact that they share a common ligand suggests that targeting only one receptor might not be effective for controlling cancer progression.

## 5. Conclusions

In conclusion, since TLR4 and AGER expressions are predominantly described in IBC tumors compared to other clinical presentations of BC, understanding IBC biology will help to improve clinical therapeutic outcomes for these patients. As shown in this study, IBC patients with obesity and highly proliferative tumors had increased percentages of TLR4 and AGER. Higher expression levels of TLR4 and AGER in IBC may contribute to the disease’s aggressiveness and serve as therapeutic target candidates. However, AGER expression negatively impacted metastasis-free survival in non-IBC subjects, suggesting that each pattern recognition receptor differentially contributes to tumor pathogenesis.

## Figures and Tables

**Table 1:** Clinicopathological data of inflammatory breast cancer (IBC) and non-IBC patients.

Parameter	IBC (n = 27) No. (%)	Non-IBC (n = 24) No. (%)	*p*-Value
Median age in years (range)	55 (36–83)	57 (32–91)	0.996
Family history of cancer			0.781
No	14 (51.9%)	11 (45.8%)	
Yes	13 (48.1%)	13 (54.2%)	
Obesity			0.304
No (BMI < 30)	18 (72.0%)	18 (85.7%)	
Yes (BMI ≥ 30)	7 (28.0%)	3 (14.3%)	
Lymph node (LN)			0.444
Negative (N0)	3 (11.1%)	5 (21.7%)	
Positive (N1/N2/N3)	24 (88.9%)	18 (78.3%)	
Metastasis			0.577
M0	14 (51.9%)	10 (41.7%)	
M1	13 (48.1%)	14 (58.3%)	
Hormone receptors			
Negative ER	8 (29.6%)	7 (29.2%)	0.999
Positive ER	19 (70.4%)	17 (70.8%)	
Negative PR	10 (37.0%)	8 (33.3%)	0.999
Positive PR	17 (63.0%)	16 (66.7%)	
HER2/neu (HER2)			
Negative HER2	22 (81.5%)	19 (79.2%)	0.999
Positive HER2	5 (18.5%)	5 (20.8%)	
Ki-67			
Ki-67 < 20%	5 (18.5%)	7 (30.4%)	0.507
Ki-67 ≥ 20%	22 (81.5%)	16 (69.6%)	
TNBC			0.731
No	21 (77.8%)	20 (83.3%)	
Yes	6 (22.2%)	4 (16.7%)	

BMI: body mass index; ER: estrogen receptor; HER2: human epidermal growth factor receptor-2; IBC: Inflammatory breast cancer; LN: lymph node; n: number of cases; PR: progesterone receptor; TNBC: triple-negative breast cancer. The statistical analysis was performed using the Chi-square test.

**Table 2:** Clinicopathological data of inflammatory or non-inflammatory breast carcinoma patients analyzed according to TLR4 and RAGE expression.

Variable	TLR4 Expression (%)			AGER Expression (%)		
	IBC (n = 17) Mean ± SD	Non-IBC (n = 15) Mean ± SD	*p*-Value	IBC (n = 18) Mean ± SD	Non-IBC (n = 16) Mean ± SD	*p*-Value
Age			0.438			0.826
<45 ^a^	99.7 ± 39.8	84.8 ± 16.9		83.7 ± 28.7	65.1 ± 36.4	
≥45 ^a^	86.7 ± 21.9	95.7 ± 58.1		112.2 ± 38.4	86.4 ± 45.1	
Family history of cancer			0.229			0.227
No	85.4 ± 25.5	77.5 ± 30.2		97.3 ± 35.6	84.3 ± 48.0	
Yes	94.7 ± 26.9	119.0 ± 61.1		117.0 ± 40.9	68.5 ± 31.4	
Obesity			0.107			0.054
No (BMI < 30 Kg/m^2^)	80.9 ± 16.8	98.4 ± 48.3		101.5 ± 34.4	86.6 ± 40.9	
Yes (BMI ≥ 30 Kg/m^2^)	112.9 ± 23.3 ^a^	74.0 ± 8.5		133.8 ± 33.0	8.2 ± 0.0	
LN status			0.072			0.814
Negative	74.3 ± 22.3	97.4 ± 50.7		141.7 ± 0.0	97.7 ± 0.0	
Positive	97.0 ± 20.2	69.9 ± 16.1		105.4 ± 38.0	75.7 ± 44.6	
Metastasis			0.656			0.386
M0	93.5 ± 32.8	97.2 ± 48.9		107.4 ± 35.0	96.6 ± 8.6	
M1	77.9 ± 12.6	96.4 ± 48.3		107.5 ± 40.7	74.2 ± 45.9	
Hormone receptor			0.538			0.309
Negative ER	81.9 ± 23.6	72.2 ± 10.4		108.4 ± 49.4	51.3 ± 36.7	
Positive ER	90.0 ± 25.5	101.0 ± 53.4		110.6 ± 38.0	90.7 ± 39.9	
Negative PR	84.0 ± 19.8	82.9 ± 18.7	0.844	98.6 ± 44.7	76.1 ± 57.4	0.705
Positive PR	91.1 ± 27.1	95.6 ± 54.7		114.1 ± 37.6	80.21 ± 29.2	
HER2			0.110			0.890
Negative	85.3 ± 21.8	96.6 ± 49.5		111.4 ± 39.6	84.4 ± 44.4	
Positive	117.1 ± 37.0	70.3 ± 12.6		92.6 ± 0.0	60.3 ± 32.4	
Ki-67			0.002 *			0.049 *
Ki67 < 20%	86.7 ± 35.1	138.2 ± 51.7		95.0 ± 33.2	96.7 ± 47.4	
Ki67 ≥ 20%	89.7 ± 22.5	67.9 ± 14.3 ^b^		121.1 ± 36.5	64.2 ± 33.5	
TNBC			0.923			0.045 *
No	92.9 ± 23.8	93.3 ± 48.4		115.5 ± 35.3	87.7 ± 40.3	
Yes	71.0 ± 25.2	74.9 ± 9.8		136.8 ± 6.8	33.6 ± 35.9	

AGER: Receptor for Advanced Glycation End Products; BMI: body mass index; ER: estrogen receptor; HER2: human epidermal growth factor receptor- 2; IBC: inflammatory breast cancer; LN: lymph node; n: number of cases; PR: progesterone receptor; TLR4: toll-like receptor 4; TNBC: triple-negative breast cancer. * Denotes a statistical difference between the IBC and non-IBC groups. The statistical analysis was performed using the two-way ANOVA and Tukey post hoc test. ^a^ *p* < 0.05, significant versus no obesity in the IBC group (Mann–Whitney test). ^b^ *p* < 0.05, significant compared with Ki67 < 20% in the non-IBC group (Mann–Whitney test).

**Table 3:** Multivariable analysis for metastasis.

Variable	*p*-Value	HR	CI95%	
Age (<50)	0.151	0.115	0.006	2.207
Radical mastectomy	***0.022*** *	***0.002***	***0.006***	***0.392***
Group (IBC vs non-IBC)	0.062	12.803	0.787	20.833
Histologic grade (III)	0.101	0.164	0.019	1.421
Clinical Stage	0.582	1.879	0.199	17.764
T	0.406	1.675	0.496	5.656
N	0.377	0.632	0.229	1.747
M	0.306	5.948	0.195	181.135
Immunohistochemistry profile	0.071	3.578	0.896	14.286
BMI	0.101	2.123	0.864	5.218
TLR4 (high)	0.043 *	***1.029***	***1.010***	***1.064***
AGER (high)	0.598	0.992	0.965	1.021
Best response to neoadjuvant chemotherapy	0.341	4.839	0.188	124.617
Neoadjvant chemotherapy	0.052	0.045	0.002	1029
Adjuvant chemotherapy	0.652	0.346	0.003	34.970
Hormone therapy	0.025 *	***0.034***	***0.002***	***0.650***

AGER: Receptor for Advanced Glycation End Products; BMI: body mass index; IBC: inflammatory breast cancer; T: tumor; N: lymph node; M: metastasis; TLR4: toll-like receptor 4. * *p* < 0.05, Cox regression for metastases. HR = hazard risk. CI95% = confidence interval for HR.

## Data Availability

The authors confirm that the data supporting the findings of this study are available within the article.

## Abbreviations

AGER	Advanced Glycation End Products Receptor
BC	Breast Cancer
BMI	Body Mass Index
IBC	Inflammatory Breast Carcinoma
TLR4	Toll-like Receptor Type 4
ER	Estrogen Receptor
PR	Progesterone Receptor
